# Improving Tuber Yield of Tiger Nut (*Cyperus esculentus* L.) through Nitrogen Fertilization in Sandy Farmland

**DOI:** 10.3390/plants13081063

**Published:** 2024-04-10

**Authors:** Xu Zheng, Jianguo Liu, Zhibo Cheng, Yingqiang Sun, Luhua Li, Jiaping Wang

**Affiliations:** 1 College of Agriculture, Shihezi University, Shihezi 832000, China; zhengxu960913@163.com (X.Z.);; 2 The Key Laboratory of Oasis Eco-Agriculture, Xinjiang Production and Construction Group, Shihezi University, Shihezi 832003, China; 3 International S&T Cooperation Base of China for Efficient Crop Production and Agricultural Environmental Protection in Oasis, Shihezi 832003, China; 4 School of Chemistry & Chemical Engineering, Anhui University, Hefei 230601, China; 5 School of Material Science & Engineering, Anhui University, Hefei 230601, China

**Keywords:** leaf functional morphology, canopy apparent photosynthesis, tuber expansion, nitrogen use efficiency

## Abstract

The cultivation of tiger nut (*Cyperus esculentus* L.) on marginal lands is a feasible and effective way to increase food production in Northern China. However, the specific influence of nitrogen fertilizer application on the growth dynamics, tuber expansion, overall yield, and nitrogen use efficiency (NUE) of tiger nuts cultivated on these sandy lands is yet to be fully elucidated. From 2021 to 2022, we conducted a study to determine the effect of N fertilizers on the leaf function morphology, canopy apparent photosynthesis (CAP), tuber yield, and NUE of tiger nut. The results indicate that the tuber yield and NUE are closely related to the specific leaf area (SLA), leaf area index (LAI), leaf nitrogen concentration per area (N_A_), CAP, and tuber expansion characteristics. Notably, significant enhancements in the SLA, LAI, N_A_, and CAP during the tuber expansion phase ranging from the 15th to the 45th day under the 300 kg N ha^−1^ treatment were observed, subsequently leading to increases in both the tuber yield and NUE. Moreover, a maximum average tuber filling rate was obtained under the N300 treatment. These improvements led to substantial increases in the tuber yield (32.1–35.5%), nitrogen agronomic efficiency (NAE, 2.1–5.3%), nitrogen partial factor productivity (NPP, 4.8–8.1%), and nitrogen recovery efficiency (NRE, 3.4–5.7%). Consequently, 300 kg N ha^−1^ of N fertilizers is the most effective dose for optimizing both the yield of tiger nut tubers and the NUE of tiger nut plants in marginal soils. Structural equation modeling reveals that N application affects the yield and NUE through its effects on leaf functional traits, the CAP, and the tuber filling characteristics. Modeling indicates that tuber expansion characteristics primarily impact the yield, while CAP predominantly governs the NUE. Above all, this study highlights the crucial role of N fertilizer in maximizing the tiger nut tuber yield potential on marginal lands, providing valuable insights into sustainable farming in dry areas.

## 1. Introduction

Tiger nut (*Cyperus esculentus* L.), known as chufa, is an underappreciated crop that is rich in nutrition and has been attracting increasing interest owing to the variety of beneficial substances in its tubers [1,2]. This product is transformed into cooking oil, flour, health supplements, and cosmetics to cater to a diverse range of human needs [3,4,5,6]. For instance, in Spain, it is utilized to manufacture beverages, whereas in Africa, it acts as an alternative to flour, addressing food insecurity [6,7]. Tiger nuts are predominantly cultivated in China as a strategic response to the scarcity of oil crop resources. As of 2022, this crop has been actively promoted and cultivated in the sandy regions of Northern China, covering an area of 24,000 ha [1]. In Africa, leading producers such as Nigeria, Niger, Mali, Senegal, Ghana, and Togo have dedicated a cumulative planting area of 20,000 ha to tiger nuts [5]. Spain holds the distinction of being the pioneering European nation to initiate the large-scale cultivation of tiger nuts, utilizing approximately 1000 ha of land for this purpose [6]. Owing to variations in regional climates and cultivation practices, the tuber yield of tiger nuts exhibits a wide range, spanning from 3000 to 8500 kg per ha [1,5,6,7]. Tiger nut plants have remarkable adaptability, a high yield, and reproductivity in less fertile areas, and they thrive in fertile soils and are arable in sandy soils. Therefore, the cultivation of tiger nut on marginal land characterized by non-arable property is a potential strategy to both enhance carbon sequestration and alleviate food shortage [8,9,10,11]. The cultivation of tiger nuts on marginal lands is of critical importance; however, sandy soils present a significant challenge to the growth of tiger nuts.

Generally, crop yield relies on both biomass accumulation and its allocation to reproductive organs, as well as the distribution of photosynthetic products from leaves in the reproductive growth phase [12,13]. Therefore, the use of N fertilizer to boost photosynthetic product accumulation at this stage is crucial for achieving high yields. Nevertheless, the photosynthesis process in crop production is dynamic [14,15]. Unlike individual leaf photosynthesis, the canopy apparent photosynthesis (CAP) rate, which integrates environmental, genetic, and population factors, provides a holistic perspective on the dynamics between crop growth and yield [12,16]. The specific leaf area (SLA) is acknowledged as a crucial measure of the leaf light capture efficiency and potential size. Additionally, factors like the leaf area index (LAI), leaf nitrogen content, and leaf tissue density (LTD) are linked to crop yield [3,17].

Tuber expanding as the final stage of tiger nut yield formation is crucial for determining the overall yield of tiger nuts [8,18]. The crop yield is correlated with the photosynthetic production capacity of leaves [19], the characteristics of tuber formation (sink), and the efficient transport and distribution of photosynthetic assimilates during the tuber enlargement phase (flow) [20]. Previous research suggests that N application can increase the aboveground photosynthetic production potential and belowground nutrient acquisition [1,21]. Yu et al. [13] showed that applying N fertilizer during the gestation and filling stages of wheat could improve leaf light and capacity and delay the weakening of leaf function. With increasing N fertilizer application, the LAI, leaf nitrogen content, and yield initially increase before stabilizing [18,21]. Current studies show that applying nitrogen fertilizer improves the canopy photosynthetic capacity, which positively impacts crop yield [13,19,22], while the way in which the sink interacts with flow dynamics under nitrogen-enriched conditions is yet to be determined.

Prior research has indicated that nitrogen fertilizer can boost the yield of tiger nut tubers on marginal lands [1]. Nonetheless, the effects of nitrogen fertilizer on the leaf functional morphology of tiger nuts, as well as the NUE and tuber swelling characteristics in response to the CAP rate, remain underexplored. The aims of this study were to investigate (1) the optimal N fertilizer application rate for cultivating tiger nuts on marginal land, (2) the influence of N on the tuber swelling characteristics and NUE, and (3) the key traits that can improve tuber swelling characteristics and increase the NUE. The outcomes of this study will provide valuable insights for growers to optimize tiger nut growth and fertilization practices in arid regions.

## 2. Materials and Methods

### 2.1. Site Description

This study was conducted in Xing’an County, Kashi city, Xinjiang Province (38°38′ N, 77°06′ E), which is located on the southern edge of the Taklimakan Desert, characterized by sandy soils (>99% sand) with a low nutrient content. For climate change data in the test area, refer to Figure S1. The physical and chemical properties of 0–20 cm soil are as follows: bulk density of 1.48 g cm^−3^; pH of 8.97; soil organic matter (SOM) value of 1.22 g kg^−1^; total nitrogen (TN) value of 0.24 g kg^−1^, total phosphorus (TP) value of 0.34 g kg^−1^, total potassium (TK) value of 8.13 g kg^−1^, available nitrogen (AN) value of 19.68 mg kg^−1^, available phosphorus (AP) value of 4.79 mg kg^−1^, and available potassium (AK) value of 50.67 mg kg^−1^.

### 2.2. Experimental Design

**Figure 1:**
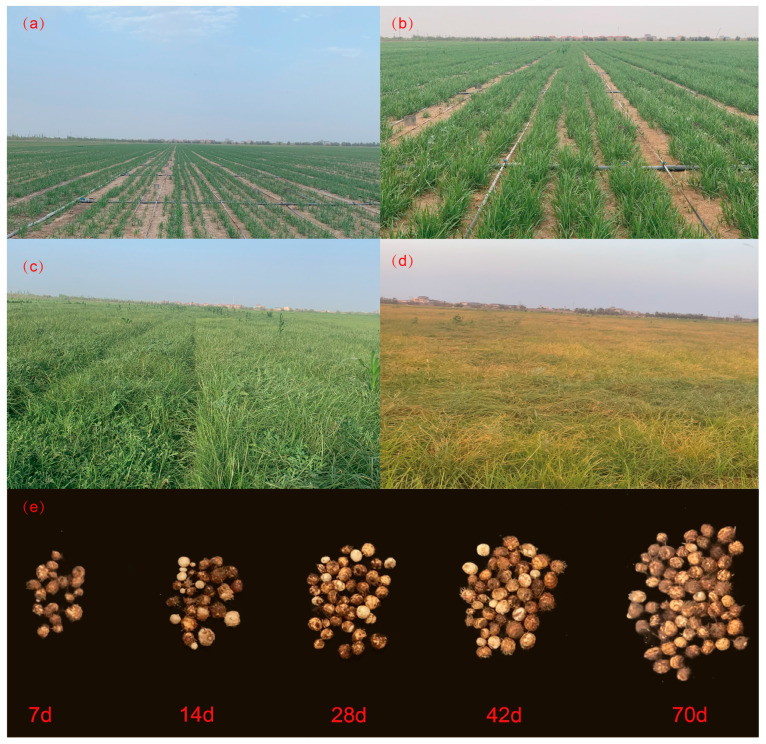
Characteristics of tiger nut in different growth stages and changes in tuber expansion stage; 10 days after planting (**a**), 40 days after planting (**b**), 70 days after planting (**c**), 110 days after planting (**d**), and different growth days in tuber expansion period (**e**).

This study was carried out with a randomized plot design, and five treatments and three replications with respective N treatments were carried out as follows: (1) no N application (N0); (2) 50% reduction in N application (100 kg N ha^−1^, N100); (3) local conventional urea—N (200 kg N ha^−1^, N200); (4) increase of 50% in N application (300 kg N ha^−1^, N300); and (5) increase of 100% in N application (400 kg N ha^−1^, N400) (Figure S2). Tiger nuts (Zhongyousha No. 1) were cultured in plots, with each plot measuring 150 m^2^ (15 × 10 m) with 2 × 0.5 m guard rows set between them. The plants were spaced 20 cm apart with a row spacing of 30 cm. During the study, weeds were regularly removed (every 15 days, manual weeding was conducted). In 2021, the tiger nuts were sown on 18 April, emerged on 4 May, and harvested on 20 September. In 2022, they were sown on 2 May, emerged on 14 May, and harvested on 10 October. Urea (N ≥ 46%, HuaJing fertilizer production company of chemical fertilizers, Aksu Prefecture, Xingjiang, China) was used as the source of N, which was applied via fertilizer dripping with water. The N0 plots only received water. Irrigation was conducted 15 times over the entire reproductive period, with each tube containing two rows spaced 60 cm apart, delivering an irrigation volume of 5250 m^3^ ha^−1^. The growth and development changes of tiger nuts during the whole growth period are shown in Figure 1.

### 2.3. Sampling and Measurement

#### 2.3.1. Canopy Apparent Photosynthesis Rate

The canopy apparent photosynthesis (CAP) rate was measured using the assimilation chamber method described by Yao et al. [12] and Liu et al. [16]. The assimilation box was 1.44 m^3^ (width 1.20 m × length 1.20 m × height 1.00 m) and made up of aluminum alloy with transparent plexiglass (transparency greater than 90%). The air in the box was blended using a 20 W electric fan. A thermometer inside the chamber was used to record the temperature.

On clear, sunny days, the CAP rates were evaluated at intervals of 0 (40 days after planting), 15 (55 days after planting), 30 (70 days after planting), 45 (85 days after planting), 60 (100 days after planting), and 70 (110 days after planting) d following the period of tuber expansion. These CAP rate evaluations were conducted from 11:00 to 13:00 under conditions where the photosynthetically active radiation above the canopy reached 1200 µmol m^–2^ s^−1^. For these measurements, an assimilation chamber was positioned over two central rows within each plot, maintaining a 30 cm gap between the rows of tiger nut. To ascertain the gas exchange rates, each plot underwent at least three measurement sessions lasting 120 s each using the LI-8100 Soil CO_2_ Flux System (LI-COR Inc., Lincoln, NE, USA). The measurement process commenced once a consistent decline in the CO_2_ levels within the chamber was observed, with concentrations varying between 400 and 430 ppm during the sampling process. The temperature inside the chamber remained within 3 °C of the external environments, and the relative humidity closely mirrored that of the ambient conditions.

Upon completing the CAP rate measurements, the plants located inside the chamber were severed at the base and extracted. Subsequently, the chamber was repositioned to its initial placement, and the assessments of gas exchange were conducted once more to ascertain the rate of soil respiration. Adjustments were made to the CAP rate readings to compensate for soil respiration.

#### 2.3.2. Leaf Functional Traits

After determining the CAP rate, the leaves were collected from all of the plants in the chamber. Leaf thickness was measured with a vernier caliper. The upper, middle, and lower parts of each leaf were measured, and the average value was considered the leaf thickness (LF). The leaf area was measured with a leaf area meter (LI-3000C Inc., Lincoln, NE, USA). The leaves were then placed in an oven (DHG-9247A, Jing Hong, Shanghai, China) and heated at 105 °C for 120 min to eliminate any greenness, followed by baking at 85 °C for at least 48 h and weighed. The leaves were dried, ground, and then analyzed using the Kjeldahl method to determine the leaf N content [1]. The leaf functional traits were calculated using the following equations [3]:

Specific leaf area (SLA, cm^2^ g^1^) = leaf area/leaf dry weigh (1)

Area-based N content (N_A_, g m^−2^) = leaf N content/leaf area (2)

Leaf tissue density (LTD, g cm^−3^) = leaf dry weigh/(leaf area × leaf thickness) (3)

Leaf area index (LAI) = leaf area/land area(4)

#### 2.3.3. Tuber Expansion

Relatively consistent plants were selected during the tuber expansion period. One hundred plants were marked with a tag in each plot, and five tagged plants were collected at 7-day intervals starting from the tuber expansion period to maturity (Figure 1e). During each sampling period, the tubers were dried and weighed. Richard’s [23] equation was used for fitting according to the methods described:

*W* = *A*/(1 + *Be*^−*kt*^)^1/*N*^ (5)

*G* = *AkBe*^−*kt*^/*N* (1 + *Be*)^(*N*+1)/*N*^ (6)

where *W* is the tuber weight (g); *A* is the final tuber weight; *t* is the time that the tuber expands (d); *B*, *k*, and *N* are the equation parameters; and *G* is the tuber expansion rate (g plant^–1^ d^–1^).

#### 2.3.4. Nitrogen Content

A total of 2.00 m^2^ of plants in each plot was sampled at the full maturity stage. The plant samples were divided into three parts (leaves, roots, and tubers), dried to a constant weight at 80 °C, and then weighed. The samples were milled and passed through a 0.5 mm sieve. After digestion in concentrated H_2_SO_4_ with a fixed N catalyst, an automatic Kjeldahl apparatus (FOSS-8400, Hillerød, Denmark) was used to determine the leaves’, roots’, and tubers’ N contents according to the Kjeldahl method [1].

#### 2.3.5. Yield and Yield Components

During the mature period, a 2 m^2^ plot was selected to represent the average yield of each plot. The tubers were manually excavated and transported to the laboratory to remove pebbles and other impurities. Subsequently, they were washed and weighed. The harvested tiger nut tubers were then placed in an oven and heated at 105 °C for 40 min to eliminate any greenness, followed by baking at 75 °C until they reached a constant weight. The theoretical yield of each community was calculated based on their areas.

### 2.4. Indicator Calculation

#### 2.4.1. Nitrogen Utilization

The N harvest index (NHI), N grain production efficiency (NGPE), N agronomic efficiency (NAE), N partial factor productivity (NPP), and N recovery efficiency (NRE) were calculated according to the methods described by Sun et al. [23]:

NHI (%) = N accumulation in tuber at maturity/total N accumulation at maturity × 100(7)

NGPE (kg kg^−1^) = tuber yield/total N accumulation at maturity (8)

NAE (kg kg^−1^) = (tuber yield in N supply − tuber yield in zero N supply)/N supply rate (9)

NPP (kg kg^−1^) = tuber yield in N supply/N supply rate (10)

NRE (%) = (total N accumulation in N supply at maturity − total N accumulation in zero N supply at maturity)/N supply rate × 100(11)

#### 2.4.2. Tuber Expansion Characteristics

The initial growth potential of the tiger nut (*R*_0_), time of maximum growth rate (*T*_max_), maximum tuber expansion rate (*G*_max_), mean tuber expansion rate during the expansion stage (*G*_mean_), and tuber expansion accumulation were calculated based on the three phases (*T*_1_, *T*_2_, and *T*_99_) after flowering, as described in Equation (5). The mean tuber expansion rate (MTR) of the three expansion periods 0–*T*_1_ (early expanding), *T*_1_–*T*_2_ (middle expanding), and *T*_2_–*T*_99_ (last expanding) was calculated based on the three phases, expanding material accumulation, and the ratio of tuber expansion contributing to the A value (RGC) in each phase was calculated according to the methods of Sun et al. [23]:

*R*_0_ = *k*/*N* (12)

*T*_max_ = (1n*B* − 1n*N*)/*k* (13)

*G*_max_ = *AkBe*^−*kT*max^/*N*(1 + *Be*^-*kT*max^)^(*N*+1)/*N*^ (14)

*G*_mean_ = *Ak*/2(*N* + 2) (15)

(16) $$T_{1}=-\ln[({N^{2}}^{\;}+3N+N\;\times \;\sqrt{N^{2}+6N+5})/2B]/k$$

(17) $$T_{2}=-\ln[(N2^{\;}+3N\;\times \;N\;\times \;\sqrt{N^{2}+6N+5})/2B]/k$$

*T*_99_ = −ln [(100/99)*^N^* − 1]/*B*/*k* (18)

### 2.5. Statistical Analysis

Data analysis and graphing were conducted using Origin 2023 (OriginLab Corp., Northampton, MA, USA). The means of the treatments were assessed for significance using the least significant difference (LSD) test (*p* < 0.05). Correlation heatmaps and partial least squares path modeling were performed using R 4.2.3 (R Core Team, 2022). The pheatmap package in R was used for the correlation analysis and heatmap generation, while the plspm package was used for the partial least squares path model analysis.

## 3. Results

### 3.1. Yield and Composition of Tiger Nut

The yield and composition of tubers were notably influenced by the rate of N fertilizer application; however, neither the year nor the interaction between N fertilizer and year exhibited a significant impact on tuber yield. Compared with the local conventional urea-N (N200) treatment, an increase in N fertilizer application (N300 and N400) significantly increased the tuber yield by 32.1–35.5% (*p* < 0.05), but there was no significant difference between the tuber yields of the N300 and N400 treatments in the years of study (Table 1). The N300 treatment significantly increased the number of tillers by 26.9–28.4% and the number of tubers by 25.3–25.6%. In the present study, the 300 kg ha^−1^ N application rate was the most suitable.

### 3.2. Leaf Functional Traits

**Figure 2:**
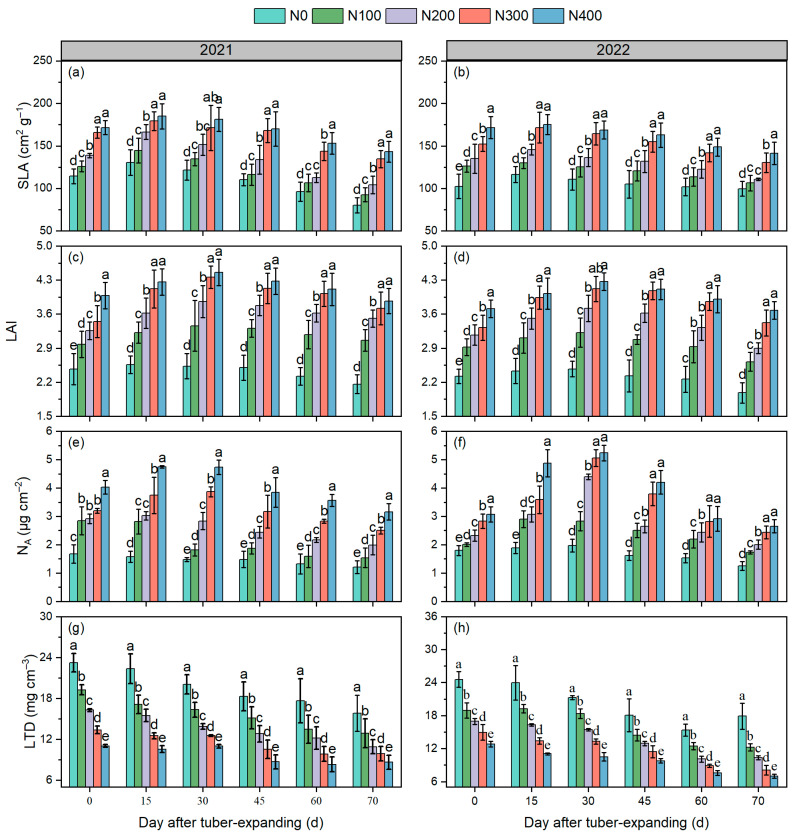
Effects of N addition on leaf functional traits in tiger nut tuber expansion. Note: SLA, specific leaf area (**a**,**b**); LAI, leaf area index (**c**,**d**); N_A_, area-based N content (**e**,**f**); LTD, leaf tissue density (**g**,**h**). Error bars indicate SD. Different lowercase letters indicate significant difference between different treatments in same period (*p* < 0.05).

**Figure 3:**
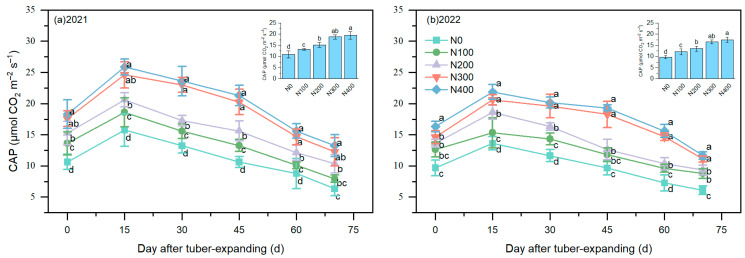
The effects of N addition on the canopy apparent photosynthetic rate during the tuber expansion period in 2021 (**a**) and 2022 (**b**) for tiger nuts. Note: The error bars indicate the SD. Different lowercase letters indicate significant differences between different treatments in the same period (*p* < 0.05).

N application had significant positive effects on key parameters, including the specific leaf area (SLA), leaf area index (LAI), area-based N content (N_A_), and leaf tissue density (LTD), during the tuber expansion stage of tiger nut (Figure 2). Nitrogen application exerted a substantial influence on the SLA, LAI, and N_A_, demonstrating a significant increase during the tuber expansion stage. Remarkably, there was no discernible distinction between the N300 and N400 treatments. As the tuber swelling progressed, the SLA for all treatments exhibited an ascending trajectory succeeded by a descending trend, peaking on the 15th day. In comparison to the N200 treatment, the SLA in the N300 and N400 treatments saw significant increases of 12.7–18.4% (2021) and 15.3–19.4% (2022), respectively. The application of nitrogen elevated the LAI during the tuber swelling stage, augmenting the photosynthetic potential. Relative to the N200 treatment, the N300 and N400 treatments displayed a substantial increase in the LAI, ranging from 16.3 to 21.4%. Conversely, the N_A_ exhibited an increase with escalating nitrogen application. In low nitrogen treatments (N0 and N100), the N_A_ demonstrated a decreasing trend over time, while in high nitrogen treatments (N300 and N400), it showcased an ascending and then descending pattern, reaching its zenith on the 30th day at 3.78–5.63 μg cm^–2^. This underscores that N application enhances the N content in the leaves during the tuber expansion stage, retards leaf senescence, and amplifies photosynthetic potential (Figure 3) and guarantees high yield (Table 1). Elevating nitrogen application significantly reduced the leaf tissue density (*p* < 0.05), with the LTD gradually diminishing as the growth stage progressed. Notably, during the tuber expansion stage, the N300 and N400 treatments exhibited a noteworthy decline in the LTD by 10.6–17.6% compared to the N200 treatment.

### 3.3. Canopy Apparent Photosynthesis Rate

N application exerted a notable impact on the CAP rate of leaves throughout the tuber expansion stage (Figure 3). This effect manifested as a discernible pattern of an initial increase followed by a subsequent decrease throughout the growth process, peaking on the 15th day of the tuber expansion stage. Remarkably, the CAP rate decreased in the N300 and N400 treatments during the 15-day to 45-day period of tuber expansion, which was markedly lower than those in the other treatments. Simultaneously, at this growth stage, the CAP rate of the overall population increased in tandem with the increasing N application rate, peaking in the N400 treatment at 14.49–19.46 μmol of CO_2_ m^−2^ s^−1^. In contrast to that in the N200 treatment, the CAP rate in the N300 and N400 treatments experienced a substantial increase of 17.86–16.42%. Although the advantages of the N300 and N400 treatments were evident, the differences between the two years were not statistically significant.

### 3.4. Tuber Expansion Characteristics

**Figure 4:**
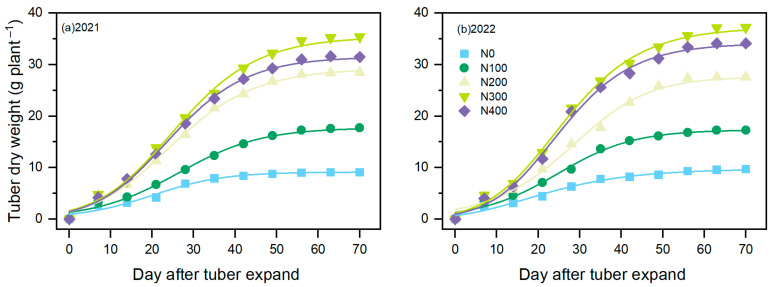
Effects of N addition on tuber expansion rate in 2021 (**a**) and 2022 (**b**) in tiger nut.

The fluctuations in the growth weight of tiger nut tubers under diverse N application rates align with those of the Richard model (Figure 4). According to the tuber expansion parameters (Table 2), the initial growth potential of the tubers (R_0_) exhibited a decreasing trend followed by an increasing trend. Notably, in 2021, the order was N400 > N300 > N200 > N0 > N100, while in 2022, it was N0 > N400 > N300 > N100 > N200. As the N application rate increased, the R0 of the tiger nut tubers initially decreased and subsequently increased, with the peak value being observed in the N400 treatment in 2021 and in the N0 treatment in 2022. The treatment that achieved the earliest *T*_max_ during tuber expansion was the N0 treatment, indicating a delayed *T*_max_ after N application. The consistent trends in the *G*_max_ and *G*_mean_ during tuber expansion under diverse N application rates suggest that the N300 treatment resulted in optimal *G*_max_ and *G*_mean_ values, followed by the N400 treatment. When examining the early swelling characteristics of tiger nut tubers (Table 3) in Figure 4, it becomes evident that the duration of tuber expansion substantially increased in the N treatments (N100, N200, N300, and N400) compared with that in the N0 treatment. The contribution rate of tuber formation to A (RGC) correlated with the days of tuber expansion and the MGR. Notably, during the initial filling stage, the N300 treatment resulted in the highest RGC at 0.435 g plant^−1^ d^−1^, closely followed by the N400 treatment. As illustrated in Table 3, the MGR reached its highest value during the middle stage of tuber expansion, and although the duration was short, the RGC contributions exceeded 60% across all of the treatments. Compared with those in the N200 treatment, both the N300 and N400 treatments significantly increased the MGR, ranging from 20.28 to 38.23%. Furthermore, with an increasing N fertilizer application rate, the MGR of the tubers increased and then decreased, reaching its lowest value in the N300 treatment. As the tuber expansion progressed to the late stage, the N400 treatment resulted in the highest MGR, followed by the N300 treatment. Notably, the N300 treatment resulted in a greater RGC during the early and middle stages of tuber expansion.

### 3.5. Nitrogen Use Efficiency

During a two-year investigation, the management of N fertilizer had a notable influence on the efficiency indicators related to its usage (Table 4). In contrast to the N200 treatment, the N100 treatment demonstrated varying degrees of reduction across all N fertilizer efficiency indicators, except for the NGPE. Notably, the NAE, NPP, and NRE decreased by 17.40–19.23%, 1.07–12.40%, and 12.34–15.40%, respectively. Compared with the N200 treatment, both the N300 and N400 treatments exhibited considerable increases, followed by subsequent decreases in the NGFE, NAE, NPP, and NRE. The N300 treatment notably showed the most substantial increases at 3.07–7.73%, 16.38–47.79%, 13.79–27.97%, and 9.58–14.94%, respectively. When the N fertilizer application rate exceeded that in the N300 treatment, the NGFE, NAE, and NRE significantly decreased by 8.86–13.55%, 8.88–13.58%, and 11.11–13.76%, respectively. The overall yield characteristics (Table 1) suggest that by increasing the N fertilizer application rate by 50% (N300), both the tuber yield and NUE of tiger nut plants can be significantly increased.

### 3.6. Correlations between Yield and NUE

**Figure 5:**
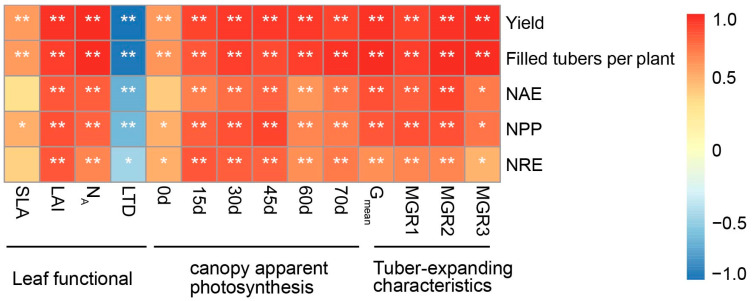
Correlation heatmap of tuber yield and NUE with leaf function, canopy apparent photosynthesis rate, and tuber-filling characteristics. Note: Coefficients of correlation between some indices (except N0) and yield and NUE (every year number of samples is 12). SLA, specific leaf area; LAI, leaf area index; N_A_, area-based N content; LTD, leaf tissue density, NAE, N agronomic efficiency, NPP, N partial factor productivity; NRE, N recovery efficiency. G_mean_, mean expansion rate tuber expansion stage; MGR, mean tuber expansion rate at certain reproductive stage; MGR1, early expansion stage; MGR2, middle expansion stage; MGR3, late expansion stage. *, *p* < 0.05; **, *p* < 0.01.

The Pearson correlation analysis (Figure 5) revealed that the SLA, LAI, and N_A_ exhibited positive correlations with tuber yield, the number of filled tubers per plant, and the NUE (R^2^ = 0.428~0.936**). Conversely, the LTD displayed a positive correlation solely with tuber yield. The number of filled tubers per plant exhibited a negative correlation with the NUE. During the tuber expansion stage from 15 to 70 days, the CAP rate was positively correlated with tuber yield, the number of filled tubers per plant, and the NUE (R^2^ = 0.523**~0.973**), specifically during the 15- to 45-day period, which was closely related to a synergistic increase in the yield and N use rate (R^2^ = 0.723**~0.973**). A significant or highly significant positive correlation was observed between the tuber expansion rate and both the yield and NUE (R^2^ = 0.654*~973**). Specifically, *G*_man_, during the early and middle expansion stages, demonstrated a noteworthy positive correlation with tuber yield and the NUE (*R*^2^ = 0.765**~0.973**), underscoring the importance of the early and middle expansion stages in tuber formation.

**Figure 6:**
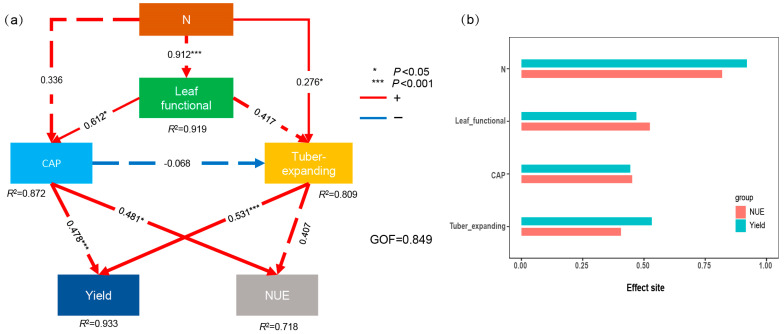
Partial least squares path model analysis of main factors and regulatory pathways affecting yield and NUE (**a**) and total effect value (**b**) of tiger nut. Leaf functional parameters (including SLA, LAI, N_A_, and LTD): CAP (0 to 70 days canopy apparent photosynthesis rate) and tuber expansion (G_mean_, early expanding stage, middle expanding stage and late expanding stage). Yield: tuber yield, NUE: NAE, NPP, and NRE.

The least squares path model revealed that N fertilizer increases tuber yield and the NUE by modulating leaf function during the tuber bulking stage and CAP rate. Overall, leaf function, the CAP, and tuber expansion accounted for 94.4% and 71.8% of the variation in tuber yield and the NUE, respectively (GOF = 0.848, Figure 6). The tuber yields primarily stemmed from the tuber expansion characteristics and CAP, with standardized direct effects of 0.531 and 0.476, respectively. Moreover, the NUE is mainly influenced by the CAP rate, with a standardized direct effect of 0.481. The total effect coefficients revealed that N fertilizer and tuber expansion had more pronounced impacts on the yield, whereas leaf function and the CAP had greater influences on the NUE. N fertilizer indirectly impacts tuber yield and the NUE by modulating the leaf function, CAP rate, and tuber expansion.

## 4. Discussion

### 4.1. Yield and Yield Formation

Numerous research efforts have consistently demonstrated that the application of N fertilizer significantly improves various critical phases of crop growth and development, leading to substantial yield increases [24,25]. The intricate process of crop yield formation is inextricably linked with several factors: population structure, photosynthetic physiology, the accumulation and transport of materials, and the components that contribute to yield [22,26]. Prior studies have established that promoting stolon tillering and shoot growth in tiger nut is essential for achieving high yields [8]. The investigations by DU et al. [4] and Asare et al. [5] revealed that for tiger nut plants to yield exceptionally high tuber yield, robust development of stolon tillering and shoots is necessary, which, in turn, facilitates the generation of a greater number of tubers within the crop population. This research highlights that the most effective N fertilizer treatment for maximizing yield primarily works by increasing the number of individual grains and stolon tillering, thereby leading to a high yield outcome (Table 1). Hence, to optimize tiger nut yield formation, a crucial strategy involves increasing the seed count per planting hole and stolon tillering while balancing the 100-seed weight of tubers [8,9]. Consequently, maintaining a steady N nutrient supply during the tuber phase and encouraging tuber formation are essential steps toward achieving a high yield.

Previous studies have demonstrated that tiger nut plants can thrive and reproduce in poor soil conditions [1,7]. Adekiya et al. [27] and Tian et al. [1] indicate that fertilization increases the aboveground biomass, tiller formation in tiger nut, the population growth rate, and dry matter accumulation. However, during tiger nut’s reproductive cycle, the tuber expansion phase is critical for tiger nut tuber yield formation. Therefore, it is essential to synchronize the development of aboveground photosynthetic substances with underground tuber formation during this phase [28]. Our study revealed that in marginal soils, the tiger nut tuber yield increases with the escalation of N application rates. Additionally, the augmentation of nitrogen fertilizer can enhance the LAI, SLA, N_A_, and CAP rate during the tuber expansion period (Figure 1 and Figure 2). This observation aligns with prior research, suggesting that optimal leaf function and the CAP rate are fundamental for high crop yields [22,24,29]. Furthermore, this study also demonstrated that the LAI, N_A_, SLA, and CAP rate during the tuber expansion period are significantly positively correlated with tiger nut yield and the number of filled tubers per plant (Figure 5). This result confirms that N fertilizer topdressing is advantageous for enhancing the photosynthetic characteristics of tiger nut plants and fostering high yields.

N fertilizer management plays a pivotal role in the formation and yield of tubers, serving as a key method for increasing the yield of tuberous crops [20,30]. Nevertheless, the effects of the timing of N fertilizer application on the yield formation process of these crops are still a matter of debate [31,32]. Various studies suggest that increasing the N and irrigation levels extends the tuber expansion period and improves stolon growth, consequently increasing tuber yield [18,21]. However, the impact of N fertilizer on the characteristics of tuber expansion has not been extensively studied. This study illustrates that, in comparison to the N200 treatment, additional N application led to an increase in R_0_ during the tuber expansion period, an earlier T_max_, and enhancements in G_max_ and G_mean_ (Figure 4; Table 4). These changes facilitate tuber expansion, accelerate the accumulation of dry matter in tubers, and ultimately increase yield [18,32]. Thus, by intensifying N fertilizer application, it is possible to reconcile the discrepancies among tuber expansion parameters (R_0_, T_max_, G_max_, and G_mean_), thereby fostering substance accumulation in tiger nut tubers and setting the stage for high yields. Faradonbeh et al. [18] and Braun et al. [21] reported that increased N fertilizer application rates increase the accumulation of substances in the tuber expansion phase, thereby increasing the yield. This study also revealed a significant positive correlation between the yield and N fertilizer application rate (Figure 5).

### 4.2. Relationship between NUE and Tuber Yield

Improving the nitrogen use efficiency not only has the potential to increase crop yields but also to reduce environmental pollution attributable to residual nitrogen in the soil [33,34]. Tian et al. [1] found that nitrogen fertilizer application could boost the aboveground biomass and specific leaf area, enhance nutrient absorption, and increase the count of tiger nut rhizomes. Our study shows that, in comparison to the N200 treatment, elevating the 400 kg N ha^−1^ markedly augmented stolon tillering and the quantity of tubers per plant (Table 1). Moreover, increment in nitrogen fertilizer input enhances nitrogen utilization efficiency metrics such as the NAE, NPP, and NRE, while it reduces four indicators, including the NHI and NGPE (Table 3). This reduction could have stemmed from the heightened nitrogen availability, which extended the tuberization phase in tuberous crops and postponed rhizome tuberization, leading to lower NHI and NGPE values [20]. Consequently, we posit that the decline in the hundred-grain weight of tiger nut is attributed to the tubers’ delayed enlargement due to an increased nitrogen fertilizer input. This suggests that a moderate increase in nitrogen fertilizer can be advantageous for boosting the nitrogen fertilizer utilization efficiency and securing high yields.

Ayyub et al. [35] demonstrated that both nitrogen uptake and the N fertilizer utilization efficiency were closely linked to tuber yield and quality in tuberous crops grown in sandy soil. Tian et al. [1] showed that N application can substantially enhance the functional attributes of tiger nut leaves in marginal soils, thus increasing tuber yield. Previous research has established a significant positive correlation between tuber yield and factors such as dry matter accumulation, leaf function, and photosynthetic capacity [22,34]. This study further confirmed the notable positive correlations between the LAI, SLA, N_A_, and CAP rate and the NAE, NPP, and NRE during the tuber expansion stage (Figure 5). However, this research focuses solely on the effects of leaf function and the CAP rate during the tuber expansion stage on tuber yield, which does not encompass the entire growth period. Moreover, there are significant variances in the ecological environment at each growth stage [20]. Future studies should aim to delineate population growth characteristics more precisely at different stages of growth.

### 4.3. Synergistically Improving the Tuber Yield and NUE

Studies have elucidated that increasing N fertilizer application during the tuber expansion stage significantly augments the CAP rate (Figure 3), elevates the photosynthetic rate and potential across the population, and promotes dry matter accumulation in the above-ground biomass, thereby facilitating tuber expansion [20,31,35]. Furthermore, an optimal N fertilizer regime can increase dry matter accumulation and increase the count of photosynthetically active leaves in tuber nut plants, increase the photosynthetic capacity, and reduce the incidence of no-tuber-producing stolons [1]. N application plays a pivotal role in harmonizing the source sink dynamics during tuber expansion, improving the assimilation of leaf-derived photosynthetic products and root N uptake, and aiding in the translocation and reallocation of carbon and N compounds to tubers [20,21]. Attaining balance between the 100-grain weight and the grain count per planting site is imperative for cultivating a high-yield population characterized by a harmoniously integrated source–sink relationship, thus increasing tuber yield and the NUE [8,36]. This methodology harbors major potential for leveraging marginal lands and achieving consistent, elevated yields of tiger nut tubers.

High photosynthetic productivity is fundamental to achieving high yield and efficient N utilization. This study revealed that N fertilizer not only markedly increased the leaf N content and LAI but also lowered the LTD (Figure 2), thus increasing the photosynthetic productivity of the crop (Figure 3). This increase could be likely attributed to N fertilizer’s role in increasing chlorophyll concentrations, expanding the photosynthetic surface area, and facilitating enhanced light penetration [19,24]. The rate at which nitrogen is translocated from leaves during the transition from the tuber enlargement stage to maturity further supports the regulatory mechanisms of tuber development, as proposed in earlier research [13,19,37]. Tuber enlargement is a crucial phase in the formation of tiger nut yield, ultimately influencing tuber quality, weight, and yield [5,8]. Currently, there is scant research on the essential pathways through which nitrogen fertilizer treatment during the tuber enlargement phase affects yield and nitrogen use efficiency, or NUE. This study indicates that leaf functional attributes during the tuber enlargement stage significantly contribute to tuber development. This study suggests that leaf functional characteristics during the tuber enlargement stage play a significant role in tuber development. Our research reveals that the tuber growth rate from days 20 to 45 of the enlargement stage accounts for more than 60% of the total enlargement period (Table 3), significantly influencing the yield and NUE. Moreover, this study demonstrates that the application of 300 kg of N ha^–1^ systematically enhances the functional and photosynthetic traits of tiger nut leaves, thereby boosting tuber yield and the NUE. Future investigations should probe into the distinct fertilizer requirements of tiger nut plants across various fertilizer formulations and delve more deeply into optimizing and controlling the release of N fertilizer in marginal soil.

## 5. Conclusions

This investigation highlights the significant influence of N application on a multitude of parameters concerning tiger nut plant cultivation in marginal soil. Specifically, N fertilizer significantly increases the LAI, SLA, NA, CAP rate, and tuber expansion parameters. Importantly, the most significant effects are observed during the early and middle expansion stages of tuber expansion, critically influencing tuber yield and the NUE (NAE, NPP, and NRE). The modulation of tiger nut tuber yield is primarily attributed to the dynamics of tuber expansion, whereas the NUE is chiefly governed by the CAP rate. Consequently, the optimization of the source–sink relationship emerges as a pivotal factor in simultaneously increasing tuber yield and the NUE. The administration of 300 kg ha^–1^ of N has emerged as an optimal approach for synergistically enhancing the source–sink dynamics of tiger nut plant in marginal soils and, as a result, substantially increasing both tuber yield and the NUE.

## Figures and Tables

**Table 1:** Effect of different fertilizer levels on yield and composition of tiger nut tuber.

Year	N Treatments	Tillers/ Number	Number of Tubers per Plant (Number)	100-Grain Weight/ g	Yield/ kg ha^−1^
2021	N0	10.3 ± 0.7 d	25.3 ± 1.7 d	64.23 ± 1.30 a	2162 ± 142 d
	N100	16.3 ± 1.2 c	42.0 ± 1.5 c	59.84 ± 1.18 b	3944 ± 184 c
	N200	19.7 ± 0.3 b	56.0 ± 4.6 b	54.32 ± 1.27 c	6026 ± 264 b
	N300	24.6 ± 0.7 a	68.0 ± 0.6 a	53.63 ± 2.79 c	7962 ± 146 ab
	N400	26.3 ± 1.2 a	72.3 ± 2.6 a	47.92 ± 1.67 d	8165 ± 247 a
2022	N0	11.3 ± 0.7 d	21.3 ± 1.5 d	61.40 ± 1.30 a	1987 ± 87 e
	N100	18.7 ± 0.9 c	43.6 ± 1.2 c	57.70 ± 0.71 b	3425 ± 124 d
	N200	22.3 ± 1.2 b	57.3 ± 2.4 b	52.30 ± 0.64 c	5856 ± 274 c
	N300	28.3 ± 0.4 a	67.0 ± 1.5 a	51.49 ± 0.41 c	7865 ± 146 ab
	N400	29.7 ± 0.8 a	70.0 ± 2.1 a	46.26 ± 0.39 d	8135 ± 172 a
Y N N × Y		0.55 ns	0.62 ns	2.27 ns	1.26 ns
		179.93 **	242.04 **	357.84 **	186.72 **
		0.45 ns	0.14 ns	0.69 ns	0.72 ns

Note: Values are shown as means ± SD. **, *p* < 0.01, ns, non-significant difference. Different lowercase letters indicate significant (*p* < 0.05) differences among treatments under same year.

**Table 2:** Effects of N addition on tuber expansion parameters of tiger nuts.

Year	N Treatments	*R*_0_	*T*_max_ (d)	*G*_max_ (g plant d^–1^)	*G*_mean_ (g plant d^–1^)
2021	N0	0.106 ± 0.02 c	20.23 ± 1.12 c	0.143 ± 0.03 c	0.174 ± 0.032 d
	N100	0.092 ± 0.08 d	26.77 ± 0.78 a	0.385 ± 0.11 c	0.290 ± 0.011 c
	N200	0.127 ± 0.012 b	24.10 ± 1.22 b	0.744 ± 0.12 a	0.517 ± 0.086 b
	N300	0.145 ± 0.12 a	24.37 ± 1.26 b	0.700 ± 0.26 a	0.624 ± 0.057 a
	N400	0.150 ± 0.08 a	23.15 ± 0.88 b	0.686 ± 0.21 b	0.588 ± 0.125 a
2022	N0	0.366 ± 0.012 a	17.09 ± 1.12 c	0.214 ± 0.033 c	0.163 ± 0.086 d
	N100	0.099 ± 0.024 d	28.95 ± 2.12 a	0.556 ± 0.126 b	0.301 ± 0.118 c
	N200	0.084 ± 0.013 d	24.82 ± 1.11 b	0.686 ± 0.138 ab	0.477 ± 0.133 b
	N300	0.190 ± 0.022 c	23.62 ± 0.88 b	0.742 ± 0.162 a	0.688 ± 0.067 a
	N400	0.317 ± 0.037 b	22.61 ± 0.37 bc	0.671 ± 0.126 ab	0.632 ± 0.126 a

Note: Values are shown as means ± SD. *R*_0_: starting growth potential of tuber; *T*_max_: time of maximum growth rate; *G*_max_: maximum filling rate during filling stage; *G*_mean_: mean filling rate during filling stage. Different lowercase letters indicate significant (*p* < 0.05) differences among treatments under same year.

**Table 3:** Effects of N addition on tuber expansion characteristics at early expansion, middle expansion, and late expansion stages of tiger nut growth.

Year	N Treatment	Early Expansion Stage			Middle Expansion Stage			Late Expansion Stage		
		Days (d)	MGR (g·plant^−1^ d^−1^)	RGC %	Days (d)	MGR (g·plant^−1^ d^−1^)	RGC %	Days (d)	MGR (g·plant^−1^ d^−1^)	RGC %
2021	N0	8.85 ± 0.62 d	0.15 ± 0.07 c	14.86 ± 1.32 d	23.96 ± 2.62 b	0.25 ± 0.11 e	65.27 ± 3.68 a	29.91 ± 2.11 c	0.06 ± 0.01 c	19.87 ± 1.42 c
	N100	13.82 ± 0.38 b	0.21 ± 0.02 b	16.24 ± 0.66 c	26.21 ± 2.24 a	0.42 ± 0.10 d	62.47 ± 0.73 ab	36.97 ± 3.42 a	0.10 ± 0.02 b	21.29 ± 0.72 b
	N200	11.73 ± 0.62 c	0.42 ± 0.11 a	17.64 ± 1.43 b	24.75 ± 1.73 ab	0.69 ± 0.18 b	60.34 ± 0.34 b	31.33 ± 2.13 b	0.20 ± 0.06 a	22.02 ± 1.15 a
	N300	17.09 ± 1.18 a	0.43 ± 0.08 a	20.62 ± 2.14 a	24.62 ± 2.24 ab	0.90 ± 0.04 a	62.27 ± 1.17 ab	28.64 ± 1.32 c	0.21 ± 0.04 a	17.11 ± 1.27 c
	N400	10.94 ± 0.87 c	0.40 ± 0.10 a	18.66 ± 0.83 ab	24.42 ± 1.43 ab	0.83 ± 0.12 ab	60.74 ± 1.98 b	27.70 ± 1.18 c	0.23 ± 0.03 a	20.60 ± 1.34 bc
2022	N0	9.24 ± 1.32 c	0.13 ± 0.03 d	12.68 ± 1.32 d	23.87 ± 2.12 b	0.27 ± 0.11 d	67.00 ± 1.32 a	30.22 ± 2.21 b	0.07 ± 0.01 d	20.32 ± 1.32 b
	N100	12.27 ± 0.72 b	0.22 ± 0.06 c	15.68 ± 0.62 c	26.72 ± 1.11 aa	0.40 ± 0.08 c	61.70 ± 1.66 b	34.87 ± 1.06 a	0.11 ± 0.02 c	22.62 ± 2.12 ab
	N200	13.36 ± 0.88 b	0.34 ± 0.11 b	16.62 ± 1.74 b	24.32 ± 1.06 b	0.68 ± 0.11 b	60.04 ± 0.68 b	31.26 ± 0.32 b	0.21 ± 0.03 b	23.34 ± 1.32 a
	N300	16.88 ± 1.26 a	0.44 ± 0.12 a	19.86 ± 1.13 a	24.46 ± 0.66 b	0.94 ± 0.12 a	61.88 ± 1.12 b	27.12 ± 1.72 c	0.25 ± 0.03 ab	18.26 ± 1.13 d
	N400	13.24 ± 1.08 b	0.42 ± 0.62 a	17.62 ± 0.32 b	25.22 ± 1.02 ab	0.85 ± 0.17 a	62.26 ± 0.43 b	26.68 ± 0.83 c	0.27 ± 0.02 a	21.12 ± 0.62 ab

Note: Values are shown as means ± SD. MGR, mean tuber expansion rate at certain reproductive stage; RGC, ratio of tuber expansion to final tuber weight. Different lowercase letters indicate significant (*p* < 0.05) differences among treatments under same year.

**Table 4:** Effects of N fertilizer on NUE of tiger nut.

Year	N Treatment	NHI (%)	NGPE (kg kg^−1^)	NAE (%)	NPP (kg kg^−1^)	NRE (%)
2021	N0	55.91 ± 3.41 a	57.00 ± 1.38 a	-	-	-
	N100	52.87 ± 1.14 ab	44.60 ± 0.60 b	9.29 ± 0.81 d	28.55 ± 2.31 b	32.56 ± 2.24 c
	N200	50.97 ± 1.99 b	38.81 ± 0.93 d	11.03 ± 0.10 c	28.86 ± 1.74 b	38.35 ± 2.55 b
	N300	50.67 ± 1.40 bc	41.81 ± 1.78 c	16.30 ± 0.47 a	36.94 ± 1.76 a	44.08 ± 1.14 a
	N400	47.17 ± 1.32 c	38.91 ± 1.45 d	14.75 ± 0.50 b	35.31 ± 0.63 a	41.79 ± 0.40 a
2022	N0	57.68 ± 1.68 a	49.46 ± 2.73 a	-	-	-
	N100	53.26 ± 2.38 b	43.85 ± 2.33 c	10.49 ± 1.24 c	30.54 ± 1.42 c	31.47 ± 0.13 c
	N200	51.43 ± 1.08 b	43.56 ± 1.66 c	12.70 ± 1.86 b	34.74 ± 2.27 b	35.90 ± 0.77 c
	N300	49.32 ± 1.85 c	44.90 ± 1.54 b	14.78 ± 0.90 a	39.53 ± 1.12 a	39.34 ± 0.87 a
	N400	47.28 ± 1.35 c	41.12 ± 1.94 cd	12.73 ± 1.28 b	36.09 ± 0.48 b	36.64 ± 1.17 b
Y		0.26	0.42	1.16	2.33	2.51
N		7.47 **	101.07 **	12.68 **	19.22 **	23.18 **
N × Y		0.14	0.33	0.12	0.69	0.48

Note: Values are shown as means ± SD. NHI, N harvest index; NGPE, N grain production efficiency; NAE, N agronomic efficiency; NPP, N partial factor productivity; NRE, N recovery efficiency. **, *p* < 0.01. Different lowercase letters indicate significant (*p* < 0.05) differences among treatments in same year.

## Data Availability

The original contributions presented in this study are included in the article; further inquiries can be directed to the corresponding author/s.

## Supplementary Materials

The following supporting information can be downloaded at https://www.mdpi.com/article/10.3390/plants13081063/s1, Figure S1: The monthly mean temperature (triangle) and precipitation (bar) of the study site in 2021 and 2022. Figure S2: Different fertilization treatments and sampling methods in study area.

## References

[1] Tan J., Wu X., He Y., Li Y., Li X., Yu X., Shi J. (2023). Mutual feedback mechanisms between functional traits and soil nutrients drive adaptive potential of tiger nuts (*Cyperus esculentus* L.) in marginal land. Plant Soil.

[2] Rebezov M., Usman Khan M., Bouyahya A., Imran M., Tufail T., Loretts O., Neverova O., Artyukhova S., Kuznetsova E., Ermolaev V. (2023). Nutritional and technical aspect of tiger nut and its micro-constituents: An overview. Food Rev. Int..

[3] Fang L., Martre P., Jin K., Du X., van der Putten P.E., Yin X., Struik P.C. (2023). Neglecting acclimation of photosynthesis under drought can cause significant errors in predicting leaf photosynthesis in wheat. Glob. Chang. Biol..

[4] Du Y., Zhang Y., Chai X., Li X., Ullah A., Islam W., Zhang Z., Zeng F. (2023). Effects of different tillage systems and mowing time on nutrient accumulation and forage nutritive value of *Cyperus esculentus*. Front. Plant Sci..

[5] Asare P.A., Kpankpari R., Adu M.O., Afutu E., Adewumi A.S. (2020). Phenotypic characterization of tiger nuts (*Cyperus esculentus* L.) from major growing areas in Ghana. Sci. World J..

[6] Pascual-Seva N., Pascual B. (2021). Determination of crop coefficient for chufa crop (*Cyperus esculentus* L. var. sativus Boeck.) for sustainable irrigation scheduling. Sci. Total Environ..

[7] Fadeyi O.J., Fabunmi T.O., Soretire A.A., Olowe V.I.O., Raphael A.O. (2023). Application of Moringa leaves as soil amendment to tiger-nut for suppressing weeds in the Nigerian Savanna. BMC Plant Biol..

[8] Tang B., Rocci K.S., Lehmann A., Rillig M.C. (2023). Nitrogen increases soil organic carbon accrual and alters its functionality. Glob. Chang. Biol..

[9] Kon’kova N.G., Khoreva V.I., Popov V.S., Yakusheva T.V., Malyshev L.L., Solovyeva A.E., Shelenga T.V. (2024). Variability of the Main Economically Valuable Characteristics of *Cyperus esculentus* L. in Various Ecological and Geographical Conditions. Plants.

[10] Kiesel A., von Cossel M., Clifton-Brown J., Lewandowski I. (2023). Valorisation of marginal agricultural land in the bioeconomy. Glob. Chang. Biol. Bioenergy.

[11] Mehmood M.A., Ibrahim M., Rashid U., Nawaz M., Ali S., Hussain A., Gull M. (2017). Biomass production for bioenergy using marginal lands. Sustain. Prod. Consum..

[12] Yao H., Zhang Y., Yi X., Zhang X., Zhang W. (2016). Cotton responds to different plant population densities by adjusting specific leaf area to optimize canopy photosynthetic use efficiency of light and nitrogen. Field Crops Res..

[13] Yu Z., Shen Z., Xu L., Yu J., Zhang L., Wang X., Yin G., Zhang W., Li Y., Zuo W. (2022). Effect of Combined Application of Slow-Release and Conventional Urea on Yield and Nitrogen Use Efficiency of Rice and Wheat under Full Straw Return. Agronomy.

[14] Huang T., Liu H., Tao J.-P., Zhang J.-Q., Zhao T.-M., Hou X.-L., Xiong A.-S., You X. (2023). Low light intensity elongates period and defers peak time of photosynthesis: A computational approach to circadian-clock-controlled photosynthesis in tomato. Hortic. Res..

[15] Burlacot A. (2023). Quantifying the roles of algal photosynthetic electron pathways: A milestone towards photosynthetic robustness. New Phytol..

[16] Liu Z., Jin W., Guo J., Yuan J., Wang S., Yang H., Meng Y., Zhou Z. (2023). Efficient cotton population photosynthetic production synergistically increases seedcotton yield and fiber quality through straw incorporation with appropriate nitrogen fertilization in wheat-cotton rotation system. Field Crops Res..

[17] Feng X., Dietze M. (2013). Scale dependence in the effects of leaf ecophysiological traits on photosynthesis: B ayesian parameterization of photosynthesis models. New Phytol..

[18] Bagheri Faradonbeh H.R., Bistgani Z.E., Barker A.V. (2022). Tuber Yield and Physiological Characteristics of Potato Under Irrigation and Fertilizer Application. Commun. Soil Sci. Plant Anal..

[19] Khan A., Yan L., Hasan M.M., Wang W., Xu K., Zou G., Liu X.-D., Fang X.-W. (2022). Leaf traits and leaf nitrogen shift photosynthesis adaptive strategies among functional groups and diverse biomes. Ecol. Indic..

[20] Da Silva A.L.B.R., Zotarelli L., Dukes M.D., van Santen E., Asseng S. (2023). Nitrogen fertilizer rate and timing of application for potato under different irrigation methods. Agric. Water Manag..

[21] Fontes P.C., Braun H., Busato C., Cecon P.R. (2010). Economic optimum nitrogen fertilization rates and nitrogen fertilization rate effects on tuber characteristics of potato cultivars. Potato Res..

[22] Simkin A.J., Faralli M., Ramamoorthy S., Lawson T. (2020). Photosynthesis in non-foliar tissues: Implications for yield. Plant J..

[23] Sun Y., Sun Y., Yan F., Li Y., Wu Y., Guo C., Ma P., Yang G., Yang Z., Ma J. (2020). Coordinating postanthesis carbon and nitrogen metabolism of hybrid rice through different irrigation and nitrogen regimes. Agronomy.

[24] Zhao W., Ren T.-H., Huang X.-Y., Xu Z., Zhou Y.-Z., Yin C.-L., Zhao R., Liu S.-B., Ning T.-Y., Li G. (2023). Leaf shape, planting density, and nitrogen application affect soybean yield by changing direct and diffuse light distribution in the canopy. Plant Physiol. Biochem..

[25] Zhang G., Liu S., Dong Y., Liao Y., Han J. (2022). A nitrogen fertilizer strategy for simultaneously increasing wheat grain yield and protein content: Mixed application of controlled-release urea and normal urea. Field Crops Res..

[26] Nie J., Li Z., Zhang Y., Zhang D., Xu S., He N., Zhan Z., Dai J., Li C., Li W. (2021). Plant pruning affects photosynthesis and photoassimilate partitioning in relation to the yield formation of field-grown cotton. Ind. Crops Prod..

[27] Adekiya A.O., Olaniran A.F., Adenusi T.T., Aremu C., Ejue W.S., Iranloye Y.M., Gbadamosi A., Olayanju A. (2020). Effects of cow dung and wood biochars and green manure on soil fertility and tiger nut (*Cyperus esculentus* L.) performance on a savanna Alfisol. Sci. Rep..

[28] Barrett R.L. (2023). Sedges on the edge: New agronomic and research opportunities?. Plant Soil.

[29] Wang Z., Wang Z., Ma L., Lv X., Meng Y., Zhou Z. (2021). Straw returning coupled with nitrogen fertilization increases canopy photosynthetic capacity, yield and nitrogen use efficiency in cotton. Eur. J. Agron..

[30] Nurmanov Y.T., Chernenok V.G., Kuzdanova R.S. (2019). Potato in response to nitrogen nutrition regime and nitrogen fertilization. Field Crops Res..

[31] Milagres C.d.C., Fontes P.C.R., Silva J.M.d. (2019). Potato tuber yield and plant morphological descriptors as affected by nitrogen application. Commun. Soil Sci. Plant Anal..

[32] Abdo A.I., Elrys A.S., Abdel-Fattah M.K., Desoky E.-S.M., Huitong L., Wang L. (2020). Mitigating nitrate accumulation in potato tubers under optimum nitrogen fertilization with K-humate and calcium chloride. J. Clean. Prod..

[33] Vilarrasa-Nogué M., Teira-Esmatges M.R., Villar J., Rufat J. (2019). Effect of N dose on soil GHG emissions from a drip-fertigated olive (*Olea europaea* L.) orchard. Sci. Total Environ..

[34] Tan Y., Xu C., Liu D., Wu W., Lal R., Meng F. (2017). Effects of optimized N fertilization on greenhouse gas emission and crop production in the North China Plain. Field Crops Res..

[35] Ayyub C., Wasim Haidar M., Zulfiqar F., Abideen Z., Wright S.R. (2019). Potato tuber yield and quality in response to different nitrogen fertilizer application rates under two split doses in an irrigated sandy loam soil. J. Plant Nutr..

[36] Da Silva A.L.B.R., Zotarelli L., Dukes M.D., Agehara S., Asseng S., van Santen E. (2018). Irrigation method and application timing effect on potato nitrogen fertilizer uptake efficiency. Nutr. Cycl. Agroecosystems.

[37] Wu C., Zhou S., Cheng X., Wei X. (2023). Alternating processes of dry and wet nitrogen deposition have different effects on the function of canopy leaves: Implications for leaf photosynthesis. Front. Plant Sci..

