# Supplementary material for: Improving Tuber Yield of Tiger Nut (Cyperus esculentus L.) through Nitrogen Fertilization in Sandy Farmland
Source: Plants (Basel). 2024 Apr 10;13(8):1063. doi: 10.3390/plants13081063 (PMC11054937; doi:10.3390/plants13081063)
Supplement: Supplementary file 1 [file plants-13-01063-s001.zip › plants-2901143-supplementary.pdf]

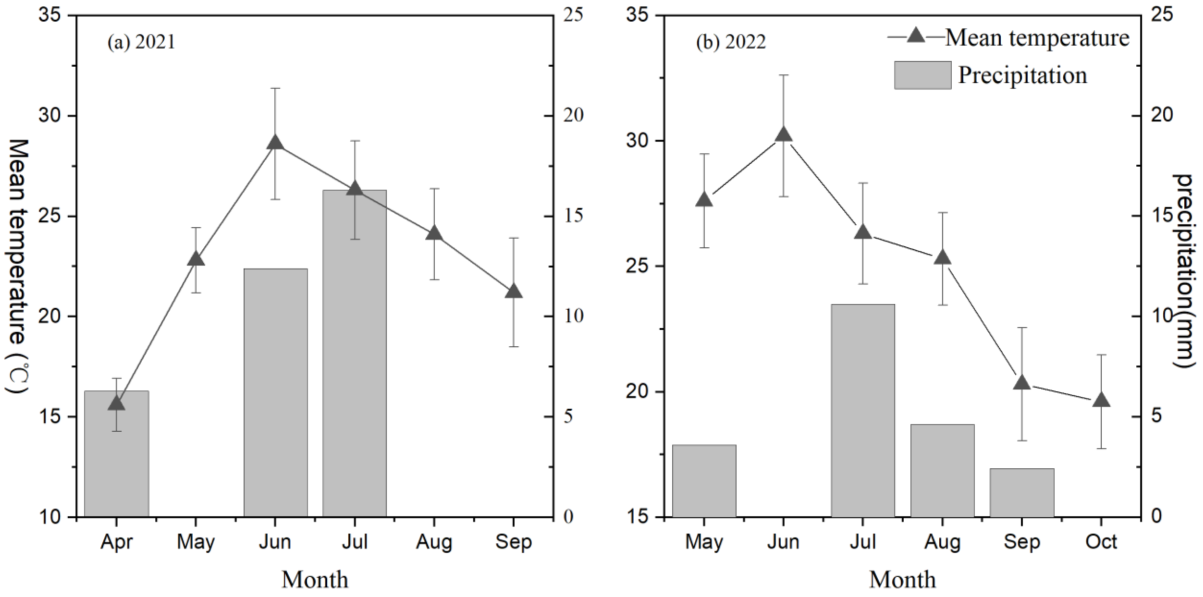

Figure S1 The monthly mean temperature (triangle) and precipitation (bar) of the study site in 2021 and 2022.

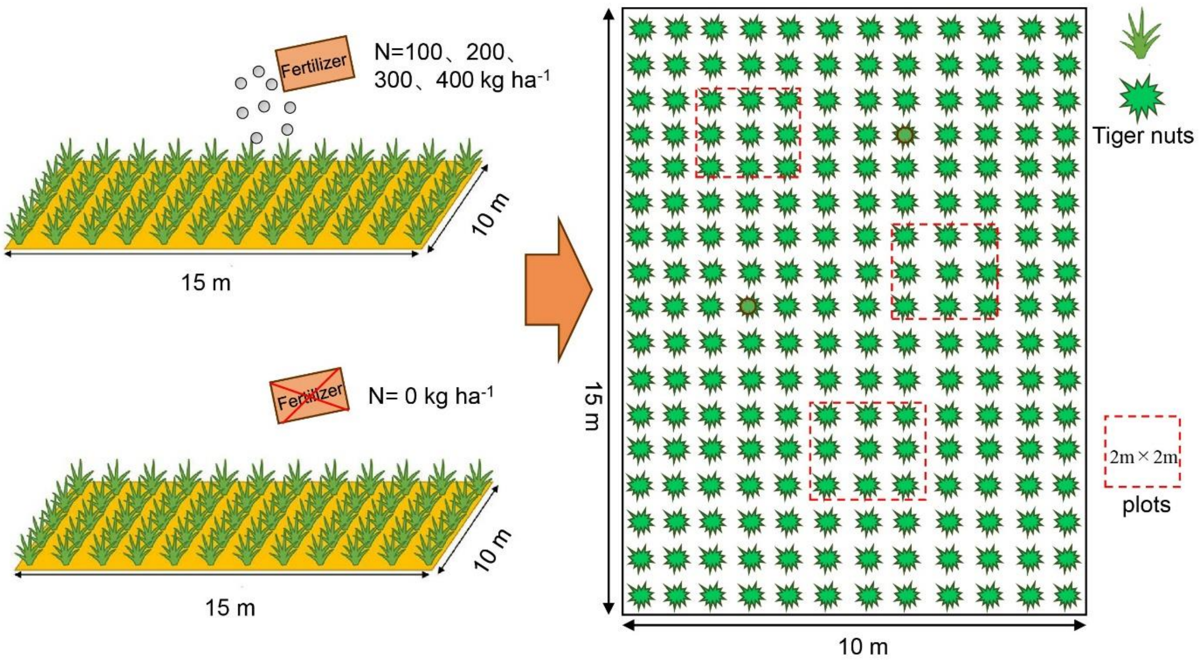

Figure S2 Different fertilization treatments and sampling methods in study area.
